# The Novel Analogue of Modafinil CE-158 Protects Social Memory against Interference and Triggers the Release of Dopamine in the Nucleus Accumbens of Mice

**DOI:** 10.3390/biom12040506

**Published:** 2022-03-27

**Authors:** Karl Ebner, Simone B. Sartori, Rita Murau, Fabian Kopel, Predrag Kalaba, Vladimir Dragačević, Johann J. Leban, Nicolas Singewald, Mario Engelmann, Gert Lubec

**Affiliations:** 1 Department of Pharmacology and Toxicology, Institute of Pharmacy and Center for Molecular Biosciences Innsbruck, University of Innsbruck, 6020 Innsbruck, Austria; simone.sartori@uibk.ac.at (S.B.S.); nicolas.singewald@uibk.ac.at (N.S.); 2 AG Neuroendokrinologie und Verhalten, Institut für Biochemie und Zellbiologie, Otto-von-Guericke-Universität Magdeburg, 39120 Magdeburg, Germany; rita.murau@med.ovgu.de (R.M.); fabian.kopel@t-online.de (F.K.); mario.engelmann@med.ovgu.de (M.E.); 3 Department of Pharmaceutical Chemistry, Faculty of Life Sciences, University of Vienna, 1010 Vienna, Austria; predrag.kalaba@univie.ac.at (P.K.); dragacevicvladimir@gmail.com (V.D.); j-e-leban@outlook.com (J.J.L.); 4 Center for Behavioral Brain Sciences, 39120 Magdeburg, Germany; 5 Department of Neuroproteomics, Paracelsus Medical University, 5020 Salzburg, Austria; gert.lubec@lubeclab.com

**Keywords:** cognitive enhancement, social recognition memory, retroactive interference, dopamine transport inhibitor, nucleus accumbens, microdialysis, long-term memory

## Abstract

Previous studies have shown that atypical dopamine-transporter-inhibitors such as modafinil and its analogues modify behavioral and cognitive functions in rodents. Here, we tested potential promnestic effects of the novel, more dopamine-transporter selective modafinil analogue CE-158 in the social discrimination memory task in male mice. Systemic administration of CE-158 1 h before the social learning event prevented the impairment of social-recognition memory following retroactive interference 3 h after the learning session of a juvenile conspecific. This effect was dose-dependent, as mice treated with 10 mg/kg, but not with 1 mg/kg CE-158, were able to discriminate between the novel and familiar conspecific despite the presentation of an interference stimulus, both 3 h and 6 h post learning. However, when 10 mg/kg of the drug was administered after learning, CE-158 failed to prevent social memory from interference. Paralleling these behavioral effects, the systemic administration of 10 mg/kg CE-158 caused a rapid and sustained elevation of extracellular dopamine in the nucleus accumbens, a brain area where dopaminergic signaling plays a key role in learning and memory function, of freely moving mice, while 1 mg/kg was not sufficient for altering dopamine levels. Taken together, our findings suggest promnestic effects of the novel dopamine-transporter-inhibitor CE-158 in a social recognition memory test that may be in part mediated via increased dopamine-neurotransmission in the nucleus accumbens. Thus, selective-dopamine-transporter-inhibitors such as CE-158 may represent interesting drug candidates for the treatment of memory complaints observed in humans with cognitive impairments and dementia.

## 1. Introduction

Memory is key for behavior and survival. Recognition memory refers to the ability to recognize a previously encountered stimulus such as a context, object, or individual. In rodents, it is evident by the spontaneous preference for exploring novelty in a variety of paradigms [1,2]. For example, their social recognition memory is based on the innate drive to investigate novel conspecifics and, thus, represent intrinsically motivated behavior [3,4,5,6,7]. Moreover, the social discrimination procedure allows for the investigation of long-term social recognition and provides an elegant experimental approach to test the durability and sensibility of social memories in mice [8,9]. Unfortunately, memories are prone to forgetting, which occurs more often during aging and in age-related neurological degenerative disorders, such as Alzheimer’s disease, and neuropsychiatric disorders, including depression, schizophrenia, and multiple sclerosis. One of the major causes of forgetting is interference phenomena [10,11,12], whereby conflicting information impairs consolidating and/or recollecting a previously acquired memory that can explain severe memory impairments in amnestic and Alzheimer’s disease with increased susceptibility to interference effects [13,14,15]. Specifically, retroactive interference involves the disruption of previously encoded information by newly learned information and, thus, may impair the consolidation of long-term memory [16,17,18]. Such an “interference stimulus” may be introduced into the social memory paradigm in rodents in order to gain insight into the cellular mechanisms underlying the generation of long-term social memory [19,20].

Several brain regions including the hippocampus, amygdala, medial prefrontal cortex, and the nucleus accumbens have been implicated in social behavior and/or social recognition memory [21,22,23]. In particular, the nucleus accumbens is a site receiving inputs from the medial prefrontal cortex, amygdala, and the hippocampus during memory processing for emotional events [24]. Furthermore, it is highly innervated by dopaminergic fibers originating in the ventral tegmental area and substantia nigra pars compacta [25,26]. The central dopamine system is well known for its role in reward processes in a context (defined behavioral parameter) and brain area-specific manner [27]. In conditioning behavioral paradigms, the relevance of local dopamine signaling for the generation of “rewards” to promote learning and memory seems easily plausible (for review [28,29]; see also [30]). However, for intrinsically rewarded learning and memory tasks such as recognition of objects and conspecifics in laboratory rodents, a modulatory role for brain dopamine signaling has also been reported [31,32,33,34]. Stimulated by studies showing that dopamine transients are induced by brief social interactions in rats [35], it has been proposed that social interaction is a highly rewarding/motivating experience that engages the dopaminergic reward circuitry [36].

Dopamine transporter inhibitors, such as the approved modafinil, enhance central dopamine signaling, but—due to the low specificity of these drugs to other monoamine neurotransmitters—cause numerous additional CNS effects. Consequently, there is great interest in synthesizing novel modafinil analogues with improved pharmacological properties, in particular with a higher selectivity towards the dopamine transporter than the noradrenaline and serotonin transporters [37,38]. Thereby, a series of modafinil derivatives have been developed in the last few years, in which the carboxy-amide moiety has been replaced with unsubstituted and substituted 5-membered ring aromatic heterocyclic moieties, yielding compounds such as CE-123 and CE-125 [38]. Through substantial structure−activity relationship optimization of CE-123, a highly specific dopamine transporter inhibitor, CE-158, was obtained. Compared to the parent compound modafinil, in CE-158, the carboxy amide moiety has been replaced with an unsubstituted thiazole ring attached through position 5, while another chiral center has been introduced through meta-bromo-substitution on a single phenyl ring. Among those, the cognitive enhancing properties of these novel modafinil derivatives are reported in various rodent memory tasks, including spatial memory and social memory interference [31,39,40,41,42,43]. For example, CE-158 has been shown to improve the cognitive flexibility and to restore learning and memory in aged rats, along with the restoration of reduced dendritic spine densities in hippocampal regions [44]. Moreover, CE-158 facilitates effort-related choice in rats [45]. Thus, while CE-158 exerts positive effects on extrinsically motivated cognitive behavior, it remains to be demonstrated whether it also affects intrinsically motivated behaviors. 

The objective of the present study was to evaluate whether CE-158 could modulate social memory and/or attenuate deficits in social recognition that is experimentally disrupted by retroactive interference. In this context, we aimed to gain insight into the potential effect of the drug to affect either acquiring and/or early consolidation of social memory and the behavior of this intrinsically motivated behavioral task. Furthermore, in vivo microdialysis was applied to investigate the effects of a single systemic administration of CE-158, similar to that used in our behavioral experiments on extracellular dopamine levels in the nucleus accumbens of freely moving mice. This brain area was chosen due to its role in the correct processing of short-term social recognition memory in rodents [22], where dopaminergic signaling seems to play a significant role [46,47,48].

## 2. Materials and Methods

### 2.1. Animals

For behavioral testing, adult male C57BL-6/OlaHSD mice (8–15 weeks old; bred in-house) were used as experimental subjects, while juveniles (25–35 days old, same background) of both sexes were used as the social stimuli. If not stated otherwise, all animals were housed in groups of 3–5 per cage (size: 20 × 37 × 15 cm) for at least 1 week before starting the experiments, under standard laboratory conditions (temperature 22 ± 1 °C, humidity 60 ± 5% with a 12/12 h light/dark cycle, lights on at 07:00 a.m., free access to food and water). For the microdialysis experiments, adult male C57BL/6J mice (Janvier-Labs, Saint Berthevin, France) were used. These animals were kept under the same conditions as the in-bred conspecifics. All experimental manipulations were approved by the Committee on Animal Health and Care of the local governmental bodies (Regierungspräsidium, Halle, Germany, registering and approval code: 42502-2-1365 UniMD; microdialysis procedures were approved by the Austrian Animal Experimentation Ethical Board; Bundesministerium für Wissenschaft, Forschung und Wirtschaft, Kommission für Tierversuchsangelegenheiten) and performed in strict compliance with the EEC recommendations for the care and use of laboratory animals (2010/63/EU). Particular effort was taken for minimizing the number of animals used, as well as their pain and suffering during experiments.

### 2.2. Drugs and Treatment

(S, S)-CE-158 (Supplementary Figure S1) was synthesized from the Lubec laboratory (University of Vienna, Austria), as described previously [44]. On the day of the experiments, CE-158 was diluted in 10% (behavioral experiments) or 30% (microdialysis experiments) Kolliphor EL (BASF, Mannheim, Germany) and sterile 0.9% saline (Berlin Chemie AG, Berlin, Germany). The Kolliphor/saline solution was administered as the vehicle control treatment. CE-158 was administered intraperitoneally at a dose of 1 and/or 10 mg/kg in a volume of 10 mL/kg body weight. In the behavioral experiments, the animals received CE-158 only once, either at a concentration of 1 mg/kg or 10 mg/kg using a cross-over design. In the microdialysis experiments, CE-158 treated animals received the drug twice via two injections (1 mg/kg and 10 mg/kg) separated by a time interval of 2 h. The dosage of the drug and the time point of administration were selected based on its relative affinity for the dopamine transporter and according to previous studies [44].

### 2.3. Behavioral Testing in the Social Discrimination Task

**Figure 1:**
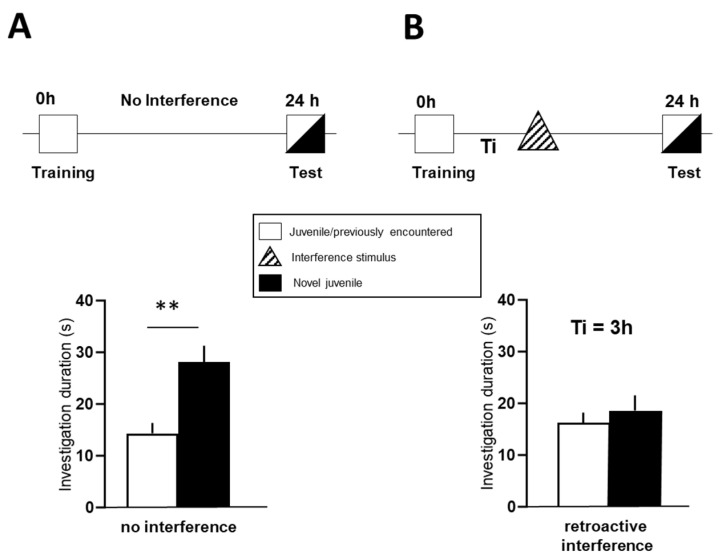
Experimental procedure for retroactive interference with juvenile recognition memory. Standard procedure for social discrimination memory (**A**) and modified procedure for testing the impact of a potentially interfering stimulus (hatched triangle) on juvenile recognition memory (**B**). Training (learning) session and “interference stimulus”-exposure were separated by a defined time interval (Ti; 3 h or 6 h). In all cases, juvenile recognition memory was assessed 24 h after training during a 4-min test session. The graphs below show intact social memory without an interference session (no interference, **left**) and impaired social memory after exposure of an interfering stimulus juvenile (retroactive interference, **right**). Data are shown as means + SEM. ** *p* < 0.01 novel vs. familiar social stimulus, as analyzed by the paired Student’s *t*-test.

The social discrimination test was modified by inducing retroactive memory interference according to previous protocols [31,49]. Initially, experimental subjects were isolated in a small polycarbonate cage (size: 14 × 20 × 15 cm) with fresh bedding for at least 2 h before the start of the experiment. In the learning (training) session, an unknown juvenile was presented to the experimental subject in the home cage of the latter. In the memory retrieval test (choice) session performed 24 h later, this familiar juvenile together with a novel juvenile were exposed to the experimental subject (Figure 1A and Supplementary Figure S2). During both the training and test session, the time spent in investigatory behavior, including sniffing and licking, towards the juvenile(s) was measured by a trained observer in a treatment-blinded manner (for details, see [49]. In addition, aggressive and sexual behaviors were monitored (Supplementary Table S2). A significant longer investigation of the novel over the familiar subject during the test session indicated an intact social recognition memory [4,49]. Recognition interference was introduced by presenting another novel juvenile (different from both the familiar and novel conspecific in test session) to the experimental animal for 4 min in the test cage 3 h or 6 h after the training session (Figure 1B).

**Figure 2:**
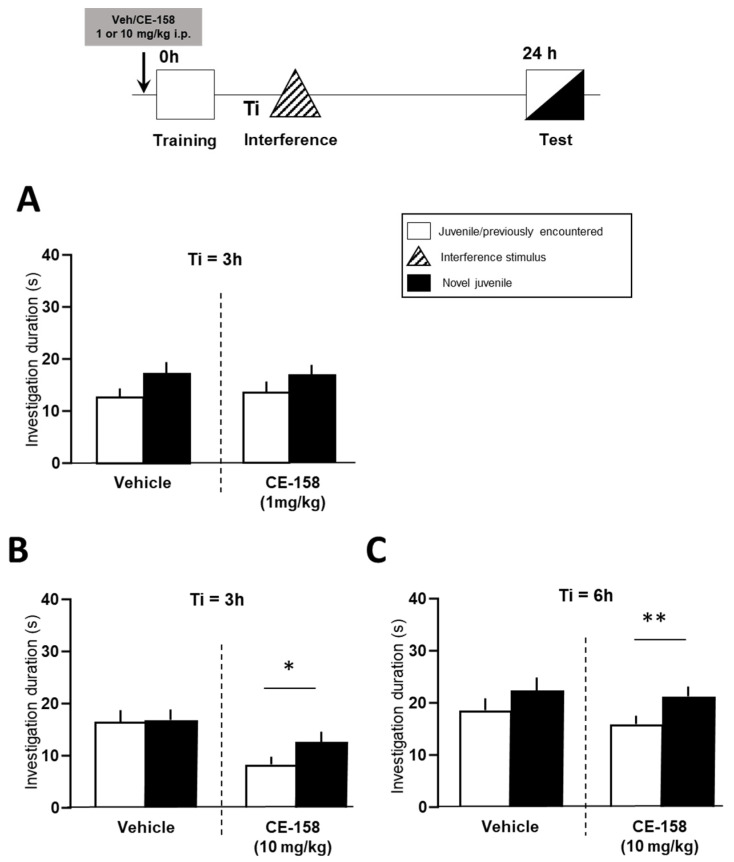
Effect of a single pre-training administration of CE-158 on deficient social memory in mice following retroactive interference. Animals were injected before training with either vehicle or CE-158 at a dose of 1 (**A**) or 10 mg/kg (**B**,**C**). In the test (choice) session, the recognition memory was assessed by exposing the experimental animal to two stimulus juveniles, the familiar one encountered in the training session and a novel one. While 1 mg/kg CE-158 did not affect memory interference (**A**), the higher dose of CE-158 (10 mg/kg) reversed the experimentally induced interference 3 h (**B**) and 6 h (**C**) after learning. Data are shown as means + SEM. * *p* < 0.05 and ** *p* < 0.01 novel vs. familiar social stimulus as analyzed by a paired Student’s *t*-test.

**Figure 3:**
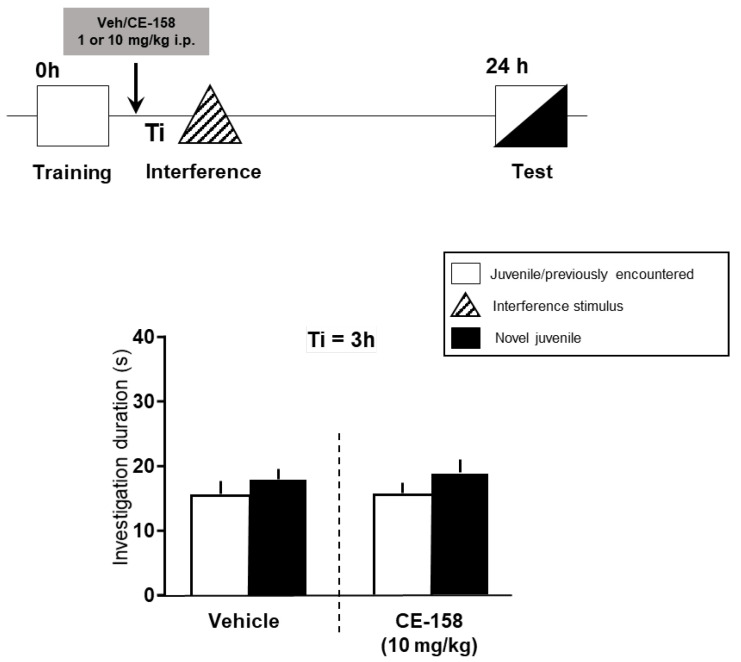
Effect of a single post-training administration of CE-158 on deficient social memory in mice following retroactive interference. Animals were injected after training with either vehicle or CE-158 at a dose of 10 mg/kg. In the test (choice) session, the recognition memory was assessed by exposing the experimental animal to two stimulus juveniles, the familiar one encountered in the training session and a novel one. The graph shows that the post learning administration of CE-158 failed to block interference, as drug treated animals were not able to discriminate between novel and familiar juvenile in the test session. Data are shown as means + SEM.

In the social memory discrimination test, a randomly assigned and balanced cross-over design, i.e., each experimental subject received both vehicle and drug in two sessions separated by at least one week, was used in order to reduce the number of animals needed according to the 3Rs. Thus, each animal was used as its own internal control. The vehicle or drug was administered either 60 min before or 60 min after the training session (Figure 2 and Figure 3).

### 2.4. In Vivo Microdialysis in Freely Moving Mice

**Figure 4:**
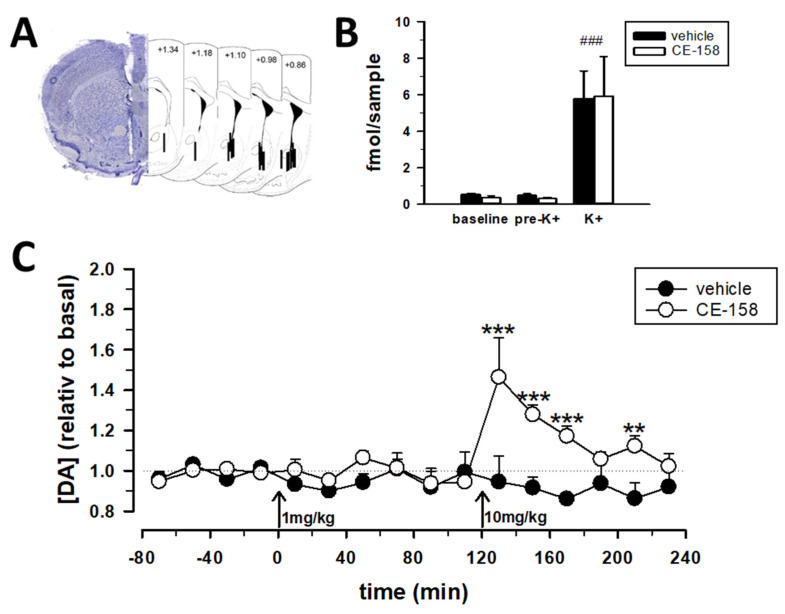
Effect of systemic administration of CE-158 on extracellular dopamine levels in the nucleus accumbens of freely moving mice. (**A**) Representative image and overview of the placement of microdialysis probes throughout the nucleus accumbens in schematic diagrams of coronal mouse brain sections (in mm from bregma). (**B**) A final local stimulation with high potassium (100 nM; K+) increased dopamine levels compared to the baseline and pre-K+ levels. (**C**) Dynamic changes in the extracellular dopamine levels after the administration of CE-158 at a dose of 1 mg/kg followed by 10 mg/kg. The arrows denote the time point of the systemic drug administration. Data are means ± SEM. *n* = 8–10 per experimental group. ** *p* < 0.01 and *** *p* < 0.001 CE-158 vs. vehicle and ### *p* < 0.001 K+-stimulation vs. baseline and pre-K+-stimulation. Analyzed by two-way ANOVA with repeated measures and a post Fisher‘s LSD test.

Under isoflurane anesthesia (at 5% and 1.5% for induction and maintenance, respectively), a commercially available guide cannula (MAB 4.15.IC with OD 0.48 mm, ID 0.35 mm, Microbiotech, Stockholm, Sweden) was implanted above the right nucleus accumbens (AP: +1.0 mm, ML: +0.8 mm, DV: 3.6 mm from Bregma) and closed with a dummy probe, as described previously [31,38]. After surgery, the animals received buprenorphine (0.5 mg/kg s.c.) and meloxicam (1.0 mg/kg p.o. via the drinking water) for analgesic care. They were allowed to recover for 5–7 days while being single-housed and habituated to the experimenter and experimental procedures. On the day before the experiment, the dummy stylet was replaced by a microdialysis probe (MAB 4.15.1.PES, Microbiotech, Sweden) that extended the guide cannula by 1 mm, thus, reaching into the nucleus accumbens. Microdialysis probes were connected to a microinfusion pump (CMA, Stockholm, Sweden) and a swivel-tether system via polyethylene tubing. Probes were constantly superfused with artificial cerebrospinal fluid (aCSF; in mM: 140 NaCl, 3.0 KCl, 1.2 CaCl_2_, 1.0 MgCl_2_, and 1.0 Na_2_HPO_4_, pH 7.4) at a flow rate of 0.5 µL/min overnight and 1.0 µL/min during sample collection, respectively. Microdialysis experiments started at 08:00–08:30 a.m. Microdialysis fractions were collected every 20 min in pre-cooled microtubes containing an antioxidative protection solution (in mM: 0.27 Na_2_EDTA, 100 acetic acid, and 0.0125 ascorbic acid), vortexed and immediately frozen at −80 °C until further analysis. After the collection of six baseline samples (from −120 to 0 min), the vehicle or CE-158 (1 mg/kg, i.p.) was administered at 10 mL/kg, and six samples were collected. Thereafter, a vehicle or CE-158 at a higher dose (10 mg/kg, i.p.) was injected and another six microdialysates were collected. At the end of the experiment, aCSF containing 100 mM KCl was used in order to elicit local depolarization, which served as a positive control for testing the functionality of the microdialysis system. Finally, animals were euthanized with an overdose of thiopental, decapitated, and their brains were removed. The localization of the microdialysis probe was verified by observing cresyl violet stained coronal sections (40 µm) under a light microscope (Figure 4A).

### 2.5. Quantification of Dopamine in Microdialysates

The quantification of dopamine in microdialysates was performed using a high-performance liquid chromatography (HPLC) system with electrochemical detection, as previously described [31,38]. The HPLC system consisted of a system controller (CBM-20A, Shimadzu Europe GmbH, Duisburg, Germany), degassing unit (DGU-20A3R, Shimadzu Europe GmbH) and a micro HPLC pump (LC-20AD, Shimadzu Europe GmbH) operated at a flow rate of 55 µL/min. Microdialysis samples (5 µL) were injected via autosampler (SIL-20ACHT, Shimadzu,) and separated on a C18 reversed-phase column (Inertsil ODS-3; 50 mm × 1.0 mm ID; 3 µm particle sizes, GL Sciences Inc., Tokyo, Japan) placed in a DECADE II electrochemical detector equipped with an amperometric flow cell (Antec, Zoeterwoude, The Netherlands). Detection was carried out at 35 °C with an applied potential of +460 mV vs. Ag/AgCl reference electrode. The mobile phase consisted of 93% *v/v* buffered aqueous solution (50 mM phosphoric acid, 50 mM citric acid, 2.36 mM octane-sulfonic acid, 0.1 mM Na_2_EDTA, pH adjusted to 5.6) and 7% (*v/v*) methanol. Under these conditions, the detection limit for dopamine was 0.15 fmol per sample. Instrument control and data acquisition were carried out by LabSolution chromatography software (version 5; Shimadzu, Kyoto, Japan). The actual dopamine concentrations in the microdialysates were calculated based on a linear regression equation of external calibration standards.

### 2.6. Statistics

Data are presented as means ± SEM. As the social discrimination test measures memory performance in a categorical manner, behavioral parameters were analyzed by paired *t*-tests for the presence or absence of memory. For in vivo microdialysis in freely moving mice, a two-way ANOVA with repeated-measures and a Fisher’s LSD post-hoc test, if allowed, were conducted for the statistical analysis using Statistica Software v13 (StatSoft GmbH, Hamburg, Germany). A *p*-value < 0.05 was considered as an indicator for a statistically significant effect.

## 3. Results

### 3.1. Effect of CE-158 on Retroactive Social Memory Interference

During the training session, all experimental mice showed a high interest in the juvenile conspecific (Supplementary Table S1). When the experimental mice were exposed to the familiar stimulus mouse in the presence of a novel mouse during test performed 24 h later, they spent more time investigating the novel juvenile than the juvenile that had already been encountered before (Figure 1A, left graph; t = 3.805, df = 23, *p* < 0.001), indicating intact recognition memory. However, the presentation of an interference stimulus 3 h after the training session blocked the social recognition abilities of the experimental mice, as indicated by similar investigation times for both juveniles during the test session (Figure 1B, right graph; t = 0.931, df = 23, *p* = 0.362).

As shown in Figure 2, the administration of CE-158 at a dose of 1 mg/kg before the learning (training) session did not affect the social recognition performance in mice exposed to retroactive memory interference (Figure 2A). The vehicle and CE-158 treated animals failed to recognize the juvenile encountered during learning session indicated by similar investigation durations towards both juveniles in the test session (vehicle: t = 1.620, df = 23, *p* = 0.119 and CE-158: t = 1.294, df = 23, *p* = 0.208). In contrast, the animals treated with the higher dose (10 mg/kg) of CE-158 rescued social recognition memory from retroactive interference, indicated by the fact that the investigation durations of the experimental subjects towards the already encountered vs. the novel stimulus animal differed significantly (Figure 2B; t = 2.088, df = 22, *p* = 0.049 vs. vehicle group: t = 0.1113, df = 22, *p* = 0.912). Moreover, the treatment of CE-158 at this dosage also blocked interference effects on memory performance when the time interval between the training session and interference was extended to 6 h (Figure 2C; t = 2.746, df = 23, *p* = 0.011). The vehicle-treated animals could not remember a juvenile already encountered during the learning session when an interference stimulus was presented 6 h after the initial learning session (t = 1.237, df = 23, *p* = 0.228). Thus, these findings suggest that treatment with CE-158 dose-dependently blocked the induction of retroactive social memory interference.

Systemic treatment with CE-158 immediately after the training session did not protect social recognition memory from interference (Figure 3; t = 1.958, df = 25, *p* = 0.061 vs. vehicle group: t = 1.043, df = 25, *p* = 0.307). The administration of CE-158 had no significant effects on other social behavioral parameters monitored during testing (e.g., aggressive/sexual behavior; Supplementary Table S2).

### 3.2. CE-158 Enhances Extracellular Dopamine Levels in the Nucleus Accumbens

Next, to assess the possible mechanisms underlying the promnestic effects of CE-158, we performed in-vivo microdialysis to monitor extracellular dopamine concentrations in the nucleus accumbens of mice following systemic drug administration. In the mouse nucleus accumbens, the extracellular dopamine levels were stable at an average of 0.44 ± 0.08 fmol/sample 80 min prior drug injection (Figure 4B). Drug treatment significantly affected the extracellular dopamine levels over time (time effect: F_(15,210)_ = 2.34; *p* = 0.004; drug treatment effect: F_(1,14)_ = 10.2, *p* = 0.007; drug treatment × time interaction: F_(15,210)_ = 3.44, *p* < 0.001). While CE-158 at a dose of 1 mg/kg did not alter the extracellular dopamine levels in the nucleus accumbens, the administration of 10 mg/kg caused a fast increase (+48%) in extracellular dopamine (Figure 4C). Subsequently, dopamine levels declined, constantly reaching baseline levels approximately 80 min after the drug administration. At the end of the microdialysis experiment, stimulation with high K+ caused a pronounced increase in extracellular dopamine levels in the nucleus accumbens compared with baseline and pre-K+ conditions (K+ effect: F_(2,24)_ = 14.5, *p* < 0.001; drug treatment × K+ interaction: F_(2,24)_ = 0.01, *p* = 0.993; Figure 4B) indicating that microdialysis systems were functional and that CE-158 did not exhaust dopamine reserves.

## 4. Discussion

In the present study, we demonstrate that the systemic administration of the novel, atypical dopamine transporter inhibitor CE-158 prior learning suppressed the retroactive interference phenomenon induced by a conspecific stimulus presented during the consolidation of the social recognition memory. In contrast, when CE-158 was administered after learning, it did not prevent deficits in memory consolidation following interference. These results suggest that CE-158 “stabilized/strengthened” the engram during acquisition for social recognition against interference in mice.

The present data extend recent studies investigating the promnestic effects of CE-158 in rats. So far, it has been found to normalize the spatial learning and memory deficits of aging rats following both chronic and acute treatment, to improve attentional set-shifting, and to ameliorate impaired discrimination learning and cognitive flexibility [44]. Furthermore, it reverses the pharmacologically-induced impaired motivational behavior in an effort-related task [45]. Overall, these data suggest that CE-158 exerts positive effects on incentive-based learning processes during motivational and intrinsic reward-related tasks. Similarly, CE-123, another modafinil analogue with an improved pharmacodynamic profile [37,38,44,50,51], has been recently shown to protect social recognition memory against interference induced 3 h, but not 6 h, post social learning [31,51,52], suggesting that CE-158 administered at the same dosage is able to protect the memory trace for a longer duration than CE-123. The differential efficacy of the two modafinil analogues could be explained by differences in the maximal increases, as well as by the temporal changes in central dopamine levels. Given that the administration of CE-158 does not appear to produce detectable toxic or adverse effects [31,44,45,51,52], the data so far suggest that this modafinil-analogue may represent an interesting drug for treating cognitive deficits in humans.

Notably, the effect on recognition memory appeared to be dose-dependent, as 10 mg/kg but not 1 mg/kg were effective in preventing deficits in social memory following interference. The higher dose of CE-158 was also effective when the interval between the learning and interference session was extended from 3 to 6 h, suggesting that CE-158 can stabilize a memory engram formed already during and immediately after learning. To address the question whether the promnestic effect of CE-158 was linked to the stabilization of the engram and/or the acquisition of the relevant information, we administered CE-158 also after learning (Figure 3). Then, however, CE-158 did not prevent interference induced impairments in social recognition. These findings suggest that CE-158 improved the learning processes operating at the time of information acquisition for the social signature of the “to be recognized” stimulus animal, rather than the consolidation process itself. However, it remains to be shown which processes are operative at the time of acquisition and, thus, may be strengthened by the drug. Nevertheless, it cannot be excluded that drugs applied before training might exert effects that also also during subsequent processes, including consolidation [53].

The consolidation of long-term social recognition memory is processed within defined brain areas/circuits including the hippocampus, medial prefrontal cortex, and nucleus accumbens [32,47,48,54]. Specifically, activity dynamics in projections from the ventral tegmental area to the nucleus accumbens encode and predict key features of social, but not novel object interaction [46], while social behavior is modulated via type 1 dopamine receptors in the nucleus accumbens [46]. On the other hand, projections from an ensemble of CA1 neurons that was preferentially reactivated in the presence of a previously encountered conspecific compared with an unfamiliar conspecific to the nucleus accumbens are necessary for social discrimination [55]. Finally, inhibition of prefrontal social-associated neurons projecting to the nucleus accumbens impairs social recognition, whereas reactivation of their projections enables recall of a previously encountered but “forgotten” conspecific [56] (see also [57]). Accumbal dopaminergic neurotransmission has been shown to be implicated in various cognitive processes including spatial memory, taste, and object recognition and social memory [22,58,59,60]. Dopamine transients are induced by brief social interactions in both adult and adolescent rats; however, adult dopamine responses habituate, while adolescent dopamine responses persist in subsequent peer interactions [35]. It has been proposed that social interaction itself is a highly rewarding/motivating experience that engages the dopaminergic reward circuitry, perhaps especially during adolescence [36].

Stimulated by these studies, we measured the dynamics in extracellular dopamine levels in the nucleus accumbens of mice after the systemic administration of CE-158. While the low dose of 1 mg/kg did not affect dopamine levels, 10 mg/kg produced a rapid increase in extracellular dopamine concentrations within 20 min following administration that remained elevated for 80 min. The temporal curve is in line with those obtained in the hippocampus of rats, as well as in pharmacokinetic studies [44]. The accumbal dopamine dynamics following the systemic administration of CE-158 in mice (Figure 4C) parallels that reported previously in rats [44,45], indicating that the impact of CE-158 on accumbal dopamine release is similar in both species. Interestingly, a similar observation of an improved memory performance, which is accompanied by an increased extracellular dopamine levels in the nucleus accumbens has been found previously in experiments using CE-123, another atypical dopamine uptake inhibitor [31,41,51]. Both compounds showing higher activities on the dopamine transporter and a higher selectivity toward this transporter vs. serotonin and norepinephrine transporters than the parent compound modafinil [37,38,44,50,51]. This improved pharmacological profile was achieved by substituting modafinil’s amide moiety by five- and six-membered aromatic heterocycles [37,38,44]. Pharmacokinetic studies showed that CE-158 penetrates the blood−brain barrier and reaches its site of action in the brain within 30 min after intraperitoneal administration in rats [44]. Moreover, CE-158 demonstrated an apparent half-life of 60–70 min and was detectable up to 10 h in the brain and plasma following a single intraperitoneal injection [44]. Thus, it can be assumed that CE-158 availability in the brain was sufficient to induce behavioral changes observed in our behavioral experiments and that found in previous studies [44,45]. However, the exact mechanisms underlying the observed effects of CE-158 on the formation and consolidation of social recognition memory at the molecular and anatomic levels are, at present, not fully understood.

## 5. Conclusions

In summary, we demonstrate that the recently developed atypical dopamine reuptake inhibitor CE-158 prevented deficits in social recognition memory in mice following retroactive interference in a dose-dependent manner. As the CE-158 action is closely associated with the detected changes in the extracellular concentration of dopamine, our data fit the hypothesis of a selective increase of dopamine signaling in brain areas thought to be involved in motivational behavior and learning and memory, such as nucleus accumbens. Thus, these findings provide additional support for the therapeutic potential of atypical dopamine reuptake inhibitors for the treatment of cognitive deficits associated with psychiatric and neurodegenerative disorders.

## Data Availability

The data presented in this study are available upon request from the corresponding author.

## Supplementary Materials

The following supporting information can be downloaded at: https://www.mdpi.com/article/10.3390/biom12040506/s1: Table S1: Investigation durations (mean ± SEM) during training and—if applicable—interference sessions of the data presented in Figure 1, Figure 2 and Figure 3. Table S2: A. Measured duration of aggressive/sexual behavior (mean ± SEM); B. Measured bouts of aggressive/sexual behavior (mean ± SEM). Figure S1: Chemical structure of (S, S)-CE-158. Figure S2: Experimental design and schedule of behavioral and microdialysis experiments. Figure S3: Effect of a single pre-training administration of vehicle (40% Kolliphor) (A) or CE-158 at a dose of 10 mg/kg diluted in 40% Kolliphor (B) on deficient social memory in mice following retroactive interference

## References

[1] Brown M.W., Aggleton J.P. (2001). Recognition memory: What are the roles of the perirhinal cortex and hippocampus?. Nat. Rev. Neurosci..

[2] Steckler T., Drinkenburg W.H., Sahgal A., Aggleton J.P. (1998). Recognition memory in rats—I. Concepts and classification. Prog. Neurobiol..

[3] Camats-Perna J., Engelmann M. (2017). Recognizing Others: Rodent’s Social Memories. Curr. Top. Behav. Neurosci..

[4] Engelmann M., Wotjak C.T., Landgraf R. (1995). Social discrimination procedure: An alternative method to investigate juvenile recognition abilities in rats. Physiol. Behav..

[5] Gabor C.S., Phan A., Clipperton-Allen A.E., Kavaliers M., Choleris E. (2012). Interplay of oxytocin, vasopressin, and sex hormones in the regulation of social recognition. Behav. Neurosci..

[6] Jacobs S.A., Huang F., Tsien J.Z., Wei W. (2016). Social Recognition Memory Test in Rodents. Bio-Protocol.

[7] Thor D.H., Holloway W.R. (1982). Social Memory of the Male Laboratory Rat. J. Comp. Physiol. Psychol..

[8] Richter K., Wolf G., Engelmann M. (2005). Social recognition memory requires two stages of protein synthesis in mice. Learn. Mem..

[9] Wanisch K., Wotjak C.T., Engelmann M. (2008). Long-lasting second stage of recognition memory consolidation in mice. Behav. Brain Res..

[10] Davis R.L., Zhong Y. (2017). The Biology of Forgetting-A Perspective. Neuron.

[11] Hardt O., Nader K., Nadel L. (2013). Decay happens: The role of active forgetting in memory. Trends Cogn. Sci..

[12] Sadeh T., Ozubko J.D., Winocur G., Moscovitch M. (2014). How we forget may depend on how we remember. Trends Cogn. Sci..

[13] Crawford L., Loprinzi P.D., Wisniewski T. (2019). Alzheimer’s Disease: Memory Interference and the Role of Exercise. Alzheimer’s Disease.

[14] Crocco E., Curiel R.E., Acevedo A., Czaja S.J., Loewenstein D.A. (2014). An evaluation of deficits in semantic cueing and proactive and retroactive interference as early features of Alzheimer’s disease. Am. J. Geriatr Psychiatry.

[15] Dewar M., Pesallaccia M., Cowan N., Provinciali L., Della Sala S. (2012). Insights into spared memory capacity in amnestic MCI and Alzheimer’s Disease via minimal interference. Brain Cogn..

[16] Alves M.V., Bueno O.F. (2017). Retroactive Interference: Forgetting as an Interruption of Memory Consolidation. Trends Psychol..

[17] Müller G.E., Pilzecker A. (1900). Experimentelle Beiträge zur Lehre vom Gedächtnis.

[18] Wixted J.T. (2004). The psychology and neuroscience of forgetting. Annu. Rev. Psychol..

[19] Camats-Perna J.C., Wotjak C.T., Stork O., Engelmann M. (2015). Timing of presentation and nature of stimuli determine retroactive interference with social recognition memory in mice. Physiol. Behav..

[20] Engelmann M. (2009). Competition between two memory traces for long-term recognition memory. Neurobiol. Learn. Mem..

[21] Deng X., Gu L., Sui N., Guo J., Liang J. (2019). Parvalbumin interneuron in the ventral hippocampus functions as a discriminator in social memory. Proc. Natl. Acad. Sci. USA.

[22] Ploeger G.E., Willemen A.P., Cools A.R. (1991). Role of the nucleus accumbens in social memory in rats. Brain Res. Bull..

[23] Tzakis N., Holahan M.R. (2019). Social Memory and the Role of the Hippocampal CA2 Region. Front. Behav. Neurosci..

[24] Kerfoot E.C., Williams C.L. (2018). Contributions of the Nucleus Accumbens Shell in Mediating the Enhancement in Memory Following Noradrenergic Activation of Either the Amygdala or Hippocampus. Front. Pharmacol..

[25] Brog J.S., Salyapongse A., Deutch A.Y., Zahm D.S. (1993). The patterns of afferent innervation of the core and shell in the “accumbens” part of the rat ventral striatum: Immunohistochemical detection of retrogradely transported fluoro-gold. J. Comp. Neurol..

[26] Voorn P., Jorritsma-Byham B., Van Dijk C., Buijs R.M. (1986). The dopaminergic innervation of the ventral striatum in the rat: A light- and electron-microscopical study with antibodies against dopamine. J. Comp. Neurol.

[27] Saddoris M.P., Siletti K.A., Stansfield K.J., Bercum M.F. (2018). Heterogeneous dopamine signals support distinct features of motivated actions: Implications for learning and addiction. Learn. Mem..

[28] Schultz W. (2002). Getting formal with dopamine and reward. Neuron.

[29] Wise R.A. (2002). Brain reward circuitry: Insights from unsensed incentives. Neuron.

[30] Tsai H.C., Zhang F., Adamantidis A., Stuber G.D., Bonci A., de Lecea L., Deisseroth K. (2009). Phasic firing in dopaminergic neurons is sufficient for behavioral conditioning. Science.

[31] Camats-Perna J., Kalaba P., Ebner K., Sartori S.B., Vuyyuru H., Aher N.Y., Dragacevic V., Singewald N., Engelmann M., Lubec G. (2019). Differential Effects of Novel Dopamine Reuptake Inhibitors on Interference With Long-Term Social Memory in Mice. Front. Behav. Neurosci..

[32] Garrido Zinn C., Clairis N., Silva Cavalcante L.E., Furini C.R., de Carvalho Myskiw J., Izquierdo I. (2016). Major neurotransmitter systems in dorsal hippocampus and basolateral amygdala control social recognition memory. Proc. Natl. Acad. Sci. USA.

[33] Gonzalez M.C., Rossato J.I., Radiske A., Bevilaqua L.R.M., Cammarota M. (2021). Dopamine controls whether new declarative information updates reactivated memories through reconsolidation. Proc. Natl. Acad. Sci. USA.

[34] Hotte M., Naudon L., Jay T.M. (2005). Modulation of recognition and temporal order memory retrieval by dopamine D1 receptor in rats. Neurobiol. Learn. Mem..

[35] Robinson D.L., Zitzman D.L., Smith K.J., Spear L.P. (2011). Fast dopamine release events in the nucleus accumbens of early adolescent rats. Neuroscience.

[36] Kopec A.M., Smith C.J., Ayre N.R., Sweat S.C., Bilbo S.D. (2018). Microglial dopamine receptor elimination defines sex-specific nucleus accumbens development and social behavior in adolescent rats. Nat. Commun..

[37] Kalaba P., Aher N.Y., Ilic M., Dragacevic V., Wieder M., Miklosi A.G., Zehl M., Wackerlig J., Roller A., Beryozkina T. (2017). Heterocyclic Analogues of Modafinil as Novel, Atypical Dopamine Transporter Inhibitors. J. Med. Chem..

[38] Kalaba P., Ilic M., Aher N.Y., Dragacevic V., Wieder M., Zehl M., Wackerlig J., Beyl S., Sartori S.B., Ebner K. (2020). Structure-Activity Relationships of Novel Thiazole-Based Modafinil Analogues Acting at Monoamine Transporters. J. Med. Chem..

[39] Aher Y.D., Subramaniyan S., Shanmugasundaram B., Sase A., Saroja S.R., Holy M., Hoger H., Beryozkina T., Sitte H.H., Leban J.J. (2016). A Novel Heterocyclic Compound CE-104 Enhances Spatial Working Memory in the Radial Arm Maze in Rats and Modulates the Dopaminergic System. Front. Behav. Neurosci..

[40] Hussein A.M., Aher Y.D., Kalaba P., Aher N.Y., Dragacevic V., Radoman B., Ilic M., Leban J., Beryozkina T., Ahmed A. (2017). A novel heterocyclic compound improves working memory in the radial arm maze and modulates the dopamine receptor D1R in frontal cortex of the Sprague-Dawley rat. Behav. Brain Res..

[41] Kristofova M., Aher Y.D., Ilic M., Radoman B., Kalaba P., Dragacevic V., Aher N.Y., Leban J., Korz V., Zanon L. (2018). A daily single dose of a novel modafinil analogue CE-123 improves memory acquisition and memory retrieval. Behav. Brain Res..

[42] Saroja S.R., Aher Y.D., Kalaba P., Aher N.Y., Zehl M., Korz V., Subramaniyan S., Miklosi A.G., Zanon L., Neuhaus W. (2016). A novel heterocyclic compound targeting the dopamine transporter improves performance in the radial arm maze and modulates dopamine receptors D1-D3. Behav. Brain Res..

[43] Sase A., Aher Y.D., Saroja S.R., Ganesan M.K., Sase S., Holy M., Hoger H., Bakulev V., Ecker G.F., Langer T. (2016). A heterocyclic compound CE-103 inhibits dopamine reuptake and modulates dopamine transporter and dopamine D1-D3 containing receptor complexes. Neuropharmacology.

[44] Lubec J., Kalaba P., Hussein A.M., Feyissa D.D., Kotob M.H., Mahmmoud R.R., Wieder O., Garon A., Sagheddu C., Ilic M. (2021). Reinstatement of synaptic plasticity in the aging brain through specific dopamine transporter inhibition. Mol. Psychiatry.

[45] Rotolo R.A., Kalaba P., Dragacevic V., Presby R.E., Neri J., Robertson E., Yang J.H., Correa M., Bakulev V., Volkova N.N. (2020). Behavioral and dopamine transporter binding properties of the modafinil analog (S, S)-CE-158: Reversal of the motivational effects of tetrabenazine and enhancement of progressive ratio responding. Psychopharmacology.

[46] Gunaydin L.A., Grosenick L., Finkelstein J.C., Kauvar I.V., Fenno L.E., Adhikari A., Lammel S., Mirzabekov J.J., Airan R.D., Zalocusky K.A. (2014). Natural neural projection dynamics underlying social behavior. Cell.

[47] Leblanc H., Ramirez S. (2020). Linking Social Cognition to Learning and Memory. J. Neurosci..

[48] Tanimizu T., Kenney J.W., Okano E., Kadoma K., Frankland P.W., Kida S. (2017). Functional Connectivity of Multiple Brain Regions Required for the Consolidation of Social Recognition Memory. J. Neurosci..

[49] Engelmann M., Hadicke J., Noack J. (2011). Testing declarative memory in laboratory rats and mice using the nonconditioned social discrimination procedure. Nat. Protoc..

[50] Nikiforuk A., Kalaba P., Ilic M., Korz V., Dragacevic V., Wackerlig J., Langer T., Hoger H., Golebiowska J., Popik P. (2017). A Novel Dopamine Transporter Inhibitor CE-123 Improves Cognitive Flexibility and Maintains Impulsivity in Healthy Male Rats. Front. Behav. Neurosci..

[51] Rotolo R.A., Dragacevic V., Kalaba P., Urban E., Zehl M., Roller A., Wackerlig J., Langer T., Pistis M., De Luca M.A. (2019). The Novel Atypical Dopamine Uptake Inhibitor (S)-CE-123 Partially Reverses the Effort-Related Effects of the Dopamine Depleting Agent Tetrabenazine and Increases Progressive Ratio Responding. Front. Pharmacol..

[52] Sagheddu C., Pintori N., Kalaba P., Dragacevic V., Piras G., Lubec J., Simola N., De Luca M.A., Lubec G., Pistis M. (2020). Neurophysiological and Neurochemical Effects of the Putative Cognitive Enhancer (S)-CE-123 on Mesocorticolimbic Dopamine System. Biomolecules.

[53] McGaugh J.L., Roozendaal B. (2009). Drug enhancement of memory consolidation: Historical perspective and neurobiological implications. Psychopharmacology.

[54] van der Kooij M.A., Sandi C. (2012). Social memories in rodents: Methods, mechanisms and modulation by stress. Neurosci. Biobehav. Rev..

[55] Okuyama T., Kitamura T., Roy D.S., Itohara S., Tonegawa S. (2016). Ventral CA1 neurons store social memory. Science.

[56] Xing B., Mack N.R., Guo K.M., Zhang Y.X., Ramirez B., Yang S.S., Lin L., Wang D.V., Li Y.C., Gao W.J. (2021). A Subpopulation of Prefrontal Cortical Neurons Is Required for Social Memory. Biol. Psychiatry.

[57] Park G., Ryu C., Kim S., Jeong S.J., Koo J.W., Lee Y.S., Kim S.J. (2021). Social isolation impairs the prefrontal-nucleus accumbens circuit subserving social recognition in mice. Cell Rep..

[58] Floresco S.B. (2015). The nucleus accumbens: An interface between cognition, emotion, and action. Annu. Rev. Psychol..

[59] Pennartz C.M., Berke J.D., Graybiel A.M., Ito R., Lansink C.S., van der Meer M., Redish A.D., Smith K.S., Voorn P. (2009). Corticostriatal Interactions during Learning, Memory Processing, and Decision Making. J. Neurosci..

[60] Setlow B. (1997). The nucleus accumbens and learning and memory. J. Neurosci. Res..

