# Supplementary material for: The Novel Analogue of Modafinil CE-158 Protects Social Memory against Interference and Triggers the Release of Dopamine in the Nucleus Accumbens of Mice
Source: Biomolecules. 2022 Mar 27;12(4):506. doi: 10.3390/biom12040506 (PMC9033101; doi:10.3390/biom12040506)
Supplement: Supplementary file 1 [file biomolecules-12-00506-s001.zip › biomolecules-1604754-supplementary.pdf]

Supplementary Table S1. Investigation durations (mean ± SEM) during training and – if applicable – interference sessions of the data presented in Figures 1-3.

Behavioral protocol	Treatment/Dosage	Training (s)	Interference (s)	Figure
24 h untreated	-	32.52 ± 3.16	-	1A
3 h retroactive interference	-	29.02 ± 3.18	27.69 ± 4.10	1B
3 h retroactive interference	pre-sampling vehicle (1 mg/kg)	12.18 ± 1.42	28.94 ± 5.72	2A
3 h retroactive interference	pre-sampling CE-158 1 mg/kg	12.56 ± 1.71	8.40 ± 1.14	2A
3 h retroactive interference	pre-sampling vehicle (10 mg/kg)	7.84 ± 1.51	6.96 ± 1.12	2B
3 h retroactive interference	pre-sampling CE-158 10 mg/kg	9.72 ± 1.31	7.70 ± 1.16	2B
6 h retroactive interference	pre-sampling vehicle (10 mg/kg)	14.25 ± 1.90	51.58 ± 5.89	2C
6 h retroactive interference	pre-sampling CE-158 10 mg/kg	14.60 ± 1.93	50.90 ± 7.55	2C
3 h retroactive interference	post-sampling vehicle (10 mg/kg)	34.69 ± 3.35	14.19 ± 1.55	3A
3 h retroactive interference	post-sampling CE-158 10 mg/kg	35.29 ± 2.86	11.49 ± 1.22	3A

Supplementary Table S2A. Measured duration of aggressive/sexual behavior (mean ± SEM)

Behavioral protocol	Treatment/Dosage	Training (s)	Interference	Choice (same)	Choice (novel)
24 h untreated	-	2.79 ± 0.65	-	1.55 ± 0.54	2.28 ± 0.68
3 h retroactive interference	-	6.93 ± 1.95	6.66 ± 1.02	2.87 ± 1.11	3.10 ± 0.98
3 h retroactive interference	pre-sampling vehicle (1mg/kg)	1.24 ± 0.37	2.20 ± 0.52	1.86 ± 0.57	2.06 ± 0.71
3 h retroactive interference	pre-sampling CE-158 (1 mg/kg)	0.65 ± 0.28	2.25 ± 0.66	2.26 ± 0.80	1.64 ± 0.53
3 h retroactive interference	pre-sampling vehicle (10 mg/kg)	2.07 ± 0.53	1.18 ± 0.32	3.30 ± 0.78	2.34 ± 0.59
3 h retroactive interference	pre-sampling CE-158 (10 mg/kg)	3.88 ± 0.82	4.22 ± 0.96	1.43 ± 0.36	2.43 ± 0.81
6 h retroactive interference	pre-sampling vehicle (10 mg/kg)	3.26 ± 0.77	3.12 ± 1.02	2.45 ± 0.72	2.95 ± 0.92
6 h retroactive interference	pre-sampling CE-158 (10 mg/kg)	2.48 ± 1.31	1.60 ± 0.73	2.76 ± 0.79	2.45 ± 0.87
3 h retroactive interference	post-sampling vehicle	4.06 ± 0.97	2.04 ± 0.62	2.14 ± 0.44	3.97 ± 1.14
3 h retroactive interference	post-sampling CE-158 (10 mg/kg)	5.45 ± 1.04	2.76 ± 0.70	2.26 ± 0.66	2.85 ± 0.73

Supplementary Table S2B. Measured bouts of aggressive/sexual behavior (mean ± SEM)

Behavioral protocol	Treatment/Dosage	Training	Interference	Choice (same)	Choice (novel)
24 h untreated	-	7 ± 2	-	4 ± 1	6 ± 1
3 h retroactive interference	-	7 ± 2	13 ± 2	5 ± 1	8 ± 3
3 h retroactive interference	pre-sampling vehicle (1mg/kg)	4 ± 1	5 ± 1	5 ± 1	5 ± 2
3 h retroactive interference	pre-sampling CE-158 (1 mg/kg)	2 ± 1	5 ± 1	5 ± 1	4 ± 1
3 h retroactive interference	pre-sampling vehicle (10 mg/kg)	8 ± 3	4 ± 1	9 ± 1	9 ± 3
3 h retroactive interference	pre-sampling CE-158 (10 mg/kg)	18 ± 4	1 ± 0	7 ± 2	14 ± 5
6 h retroactive interference	pre-sampling vehicle (10 mg/kg)	8 ± 2	4 ± 1	6 ± 1	7 ± 2
6 h retroactive interference	pre-sampling CE-158 (10 mg/kg)	5 ± 2	3 ± 1	6 ± 1	7 ± 2
3 h retroactive interference	post-sampling vehicle	9 ± 2	5 ± 1	6 ± 1	8 ± 2
3 h retroactive interference	post-sampling CE-158 (10 mg/kg)	10 ± 2	7 ± 2	7 ± 3	8 ± 2

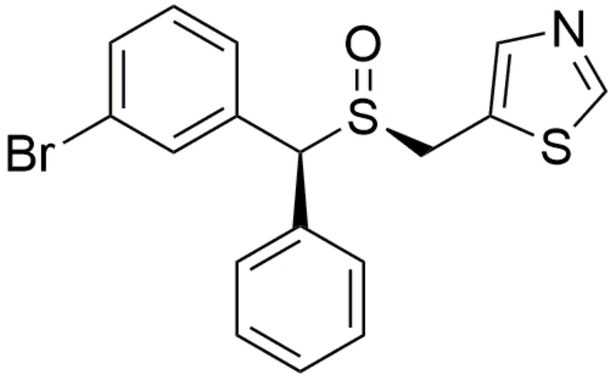

Supplementary Figure S1. Chemical structure of (S, S)-CE-158 (5-(((S)-((S)-(3-bromophenyl)(phenyl) methyl)sulfinyl)methyl)thiazole).

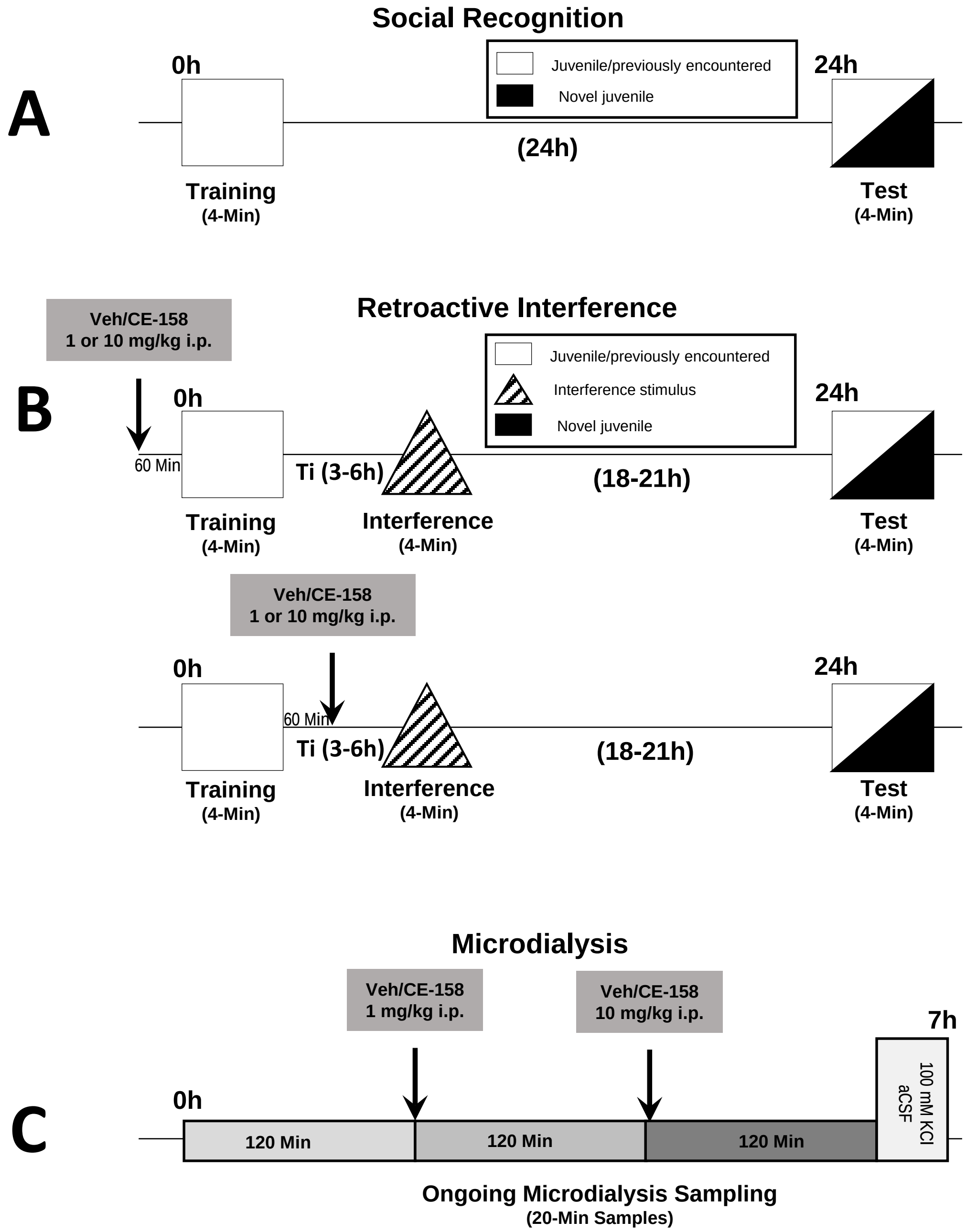

Supplementary Figure S2. Experimental design and schedule of behavioral and microdialysis experiments. Standard procedure for social discrimination memory (A) and the modified procedure with a potentially interfering stimulus (hatched triangle) for testing the impact of CE-158 on juvenile recognition memory (B). Animals were injected either 60 min before or after training with vehicle or CE-158 (at a dose of 1 or 10 mg/kg). Training and 'interference stimulus'-exposure were separated by a defined time interval (Ti; 3 or 6h). In all cases, long-term juvenile recognition memory was assessed 24h after training during a 4-min test/choice session. In the microdialysis experiments (C), CE-158 treated animals received the drug twice via two injections (1 mg/kg and 10 mg/kg) separated by a time interval of 2 hrs.

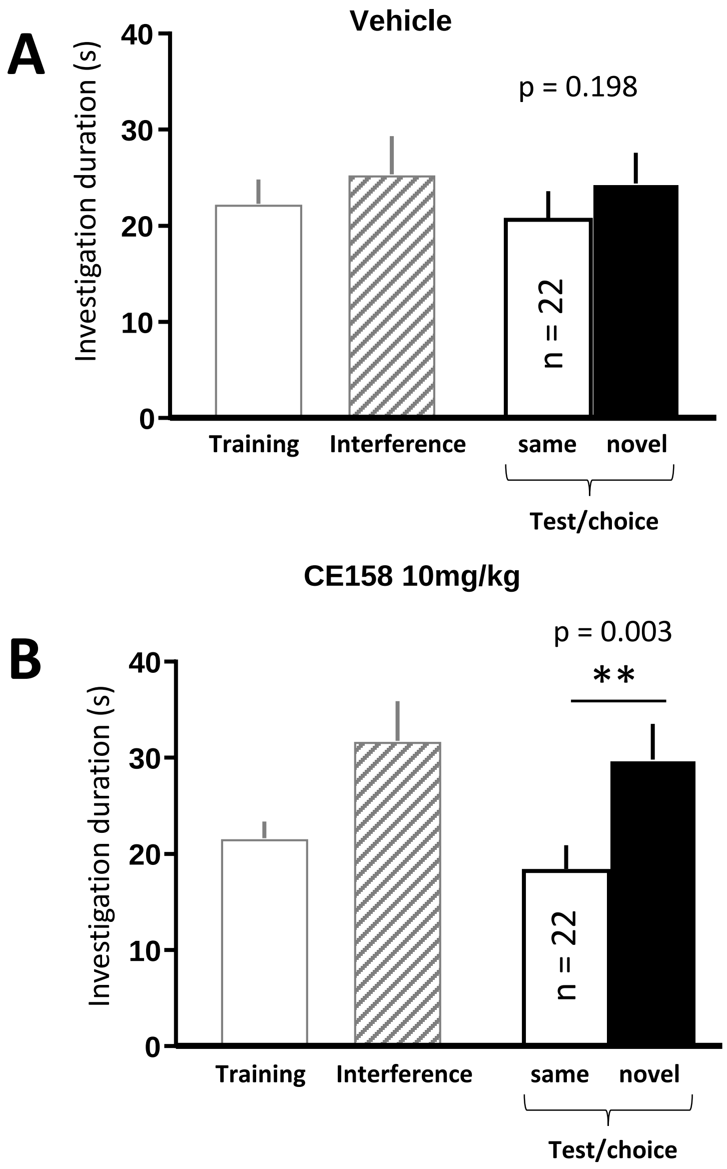

Supplementary Figure S3. Effect of a single pre-training administration of vehicle (40% Kolliphor) (**A**) or CE-158 at a dose of 10 mg/kg diluted in 40% kolliphor (**B**) on deficient social memory in mice following retroactive interference. In the test (choice) session the recognition memory was assessed by exposing the experimental animal to two stimulus juveniles, the familiar one encountered in the training session and a novel one. The graph shows a similar outcome in terms of a beneficial effect of CE-158 treatment to block the experimentally induced interference 3 h after learning. Data are shown as means + sem.
